# Supplementary material for: Klf5 regulates muscle differentiation by directly targeting muscle-specific genes in cooperation with MyoD in mice
Source: eLife. 2016 Oct 15;5:e17462. doi: 10.7554/eLife.17462 (PMC5074804; doi:10.7554/eLife.17462)
Supplement: Figure 4—source data 1. — Functional annotations associated with genes that were induced >1.5-fold with Klf5 knockdown at day 3 post differentiation. DOI: http://dx.doi.org/10.7554/eLife.17462.011 [file elife-17462-fig4-data1.doc]

GO Description % Log10 (p)

ion homeostasis 6.46 -7.19

DNA conformation change 3.34 -6.53

organelle fission 5.60 -6.18

organ morphogenesis 7.64 -6.15

recombinational repair 2.37 -6.12

epithelial mesenchymal transition 2.69 -5.70
