# Supplementary material for: Klf5 regulates muscle differentiation by directly targeting muscle-specific genes in cooperation with MyoD in mice
Source: eLife. 2016 Oct 15;5:e17462. doi: 10.7554/eLife.17462 (PMC5074804; doi:10.7554/eLife.17462)
Supplement: Supplementary file 1. — DOI: http://dx.doi.org/10.7554/eLife.17462.020 [file elife-17462-supp1.doc]

**Supplementary information**

Table 1: list of oligonucleotides

| **Gene** | **Application** | **Sense primer (5’-3’)** | **Antisense primer (5’-3’)** |
| --- | --- | --- | --- |
| *Klf5* | qPCR | GGTTGCACAAAAGTTTATAC | GGCTTGGCGCCCGTGTGCTTCC |
| *Pax7* | qPCR | AGGCCTTCGAGAGGACCCAC | CTGAACCAGACCTGGACGCG |
| *Myod1* | qPCR | GGCTACGACACCGCCTACTA | GAGATGCGCTCCACTATGCT |
| *Myog* | ChIP-qPCR | GAATCACATGTAATCCACTGGA | ACGCCAACTG CTGGGTGCCA |
| *Myog* | qPCR | AGTGAATGCAACTCCCACAG | ACGATGGACGTAAGGGAGTG |
| *Myh3* | qPCR | AAAAGGCCATCACTGACGC | CAGCTCTCTGATCCGTGTCTC |
| *Gapdh* | ChIP-qPCR and qPCR | AATGTGTCCGTCGTGGATCT | CATCGAAGGTGGAAGAGTGG |
| *Mybph* | ChIP-qPCR | CCTGCTCAGCTAATCCTTGG | CCTCCTAAGCAGCAAACAGG |
| *Mybph* | qPCR | CCTGAACCTCCGAGTGAAGAT | TCCAACACATAGCCTTGAAGC |
| *Myl4* | ChIP-qPCR | TGCCTGAACTGTTGAGCATC | GCAGCCCACTACACATTCCT |
| *Myl4* | qPCR | AAGAAACCCGAGCCTAAGAAGG | TGGGTCAAAGGCAGAGTCCT |
| *Myom2* | ChIP-qPCR | GCGTTGACTGGCTAGAGTCC | TCACACAGCATGTGTCCAAA |
| *Myom2* | qPCR | AAAAGACACAAGCACTTTGACCA | TGGGAGGATGACTGGGTGG |
| *Klf1* | qPCR | CCTGGCCTCAGACAAAGGG | ATTTCCCGTAAACTTTCTCGCA |
| *Klf2* | qPCR | CTCAGCGAGCCTATCTTGCC | CACGTTGTTTAGGTCCTCATCC |
| *Klf3* | qPCR | GAAGCCCAACAAATATGGGGT | GGACGGGAACTTCAGAGAGG |
| *Klf4* | qPCR | GCTCCTCTACAGCCGAGAATC | ATGTCCGCCAGGTTGAAG |
| *Klf6* | qPCR | GTTTCTGCTCGGACTCCTGAT | TTCCTGGAAGATGCTACACATTG |
| *Klf7* | qPCR | TCCACGACACCGGCTACTT | GGGAGCAGCAAGGGGTCTA |
| *Klf8* | qPCR | TCAGAAAGTGGTTCGATGCAG | AACAGAGCTGGGTTCTCCATT |
| *Klf9* | qPCR | TTATTGCACGCTGGTCACTATC | CTCATCGGGACTCTCCAGAC |
| *Klf10* | qPCR | GTGACCGTCGGTTTATGAGGA | AGCTTCTTGGCTGATAGGTGG |
| *Klf11* | qPCR | CATGGACATTTGTGAGTCGATCC | CCTTTGGTAGATCAGGTGCAG |
| *Klf12* | qPCR | CAGCGCCCTTGAGAACAGAAT | GTGGACGTTTGGAGACCCTTG |
| *Klf13* | qPCR | CCTGGCCTCAGACAAAGGG | ATTTCCCGTAAACTTTCTCGCA |
| *Klf14* | qPCR | CTCCGTGTGCCTCAACTAGC | CAGGCGCATCCAGGATAGC |
| *Klf15* | qPCR | GAGACCTTCTCGTCACCGAAA | GCTGGAGACATCGCTGTCAT |
| *Klf16* | qPCR | ATCCTGGCCGATCTGAGAGG | GTGCGAAGACTTGTAATAGGCT |
| *Klf17* | qPCR | AATAAGGAACAGGCTATGCACC | GTGGCTGATGAAATCCGCTG |
| *Klf5* guide RNA1 | CRSPR-Cas9 | CACCgTGCGAACCCGGCCCGCGACG | aaacCGTCGCGGGCCGGGTTCGCAc |
| *Klf5* guide RNA2 | CRSPR-Cas9 | CACCgCGGAGAAGAGCGCCGCGTCG | aaacCGACGCGGCGCTCTTCTCCGc |
| GFP guide RNA1 (Control 1) | CRSPR-Cas9 | CACCgGAGCTGGACGGCGACGTAAA | aaacTTTACGTCGCCGTCCAGCTCc |
| GFP guide RNA2 (Control 2) | CRSPR-Cas9 | CACCgAAGTTCAGCGTGTCCGGCGA | aaacTCGCCGGACACGCTGAACTTc |

Table 2: list of antibodies

| **Name** | **Application** | **Company** |
| --- | --- | --- |
| Anti-KLF5 | IF: 1/100  Western blotting: 1/1,000 | Kyowa Hakko, KM1784 |
| Anti-KLF5 | Western blotting: 1/1,000  IP, ChIP: 2 µg/sample | Santa-Cruz Biotecnology, sc-22797 RRID:AB_2130422 |
| Anti-Pax7 | IF: 1/100 | Santa-Cruz Biotecnology, sc-81648  RRID:AB_2159836 |
| Anti-MyoD1 | IF: 1/200  Western blotting: 1/1,000  IP, ChIP: 2 µg/sample | Santa-Cruz Biotechnology, sc-760  RRID:AB_2148870 |
| Anti-MyoD1 | Western blotting: 1/1,000 | DAKO, Clone 5.8A, M3512, RRID:AB_2148874 |
| Anti-Myogenin | IF: 1/200  Western blotting: 1/500 | DAKO, Clone F5D, M3559,  RRID:AB_2250893 |
| Anti-Embryonic Myosin Heavy Chain | IF: 1/50 | Developmental Studies Hybridoma Bank (DSHB), Clone F1.652, RRID:AB_528358 |
| Anti-Sarcomeric Myosin Heavy Chain | IF: 1/500  Western blotting 1:1,000 | DSHB, Clone MF20  RRID:AB_2147781 |
| Anti-Laminin | IF: 1/500 | SIGMA-ALDRICH, L9393, RRID:AB_477163 |
| Anti-Type I Collagen | IF: 1/200 | SouthernBiotech, 1310-01 |
| Anti-MEF2 (C-21) | Western blotting: 1/1,000  IP, ChIP: 2 µg/sample | Santa-Cruz Biotechnology, sc-313X, RRID:AB_631920 |
| Anti-β-Tubulin | Western blotting: 1/10,000 | Wako, 014-25041 |
| Goat anti-mouse IgG-Alexa Fluor 546 | IF: 1:20,000 | ThermoFisher Scientific, A11018,  RRID:AB_2534085 |
| Goat anti-rabbit IgG-Alexa Fluor 488 | IF: 1:10,000 | ThermoFisher Scientific, A11070, RRID:AB_10561551 |
| Goat anti-rat IgG-Alexa Fluor 488 | IF: 1: 10,000 | ThermoFisher Scientific, A11006  RRID:AB_2534074 |
| Donkey anti-goat IgG-Alexa Fluor 594 | IF: 1: 10,000 | ThermoFisher Scientific, A11058  RRID:AB_2534105 |
| Anti-mouse IgG, HRP Linked whole Ab | IF: 1: 10,000 | GE Healthcare, NA931,  RRID:AB_772210 |
| Anti-rabbit IgG, HRP-linked | IF: 1: 10,000 | Cell Signaling Technology, #7074,  RRID:AB_10697506 |
| Anti-rat IgG, HRP Linked whole Ab | IF: 1: 10,000 | GE Healthcare, NA935  RRID:AB_772207 |
